# Associations of quality of life with physical activity, fruit and vegetable consumption, and physical inactivity in a free living, multiethnic population in Hawaii: a longitudinal study

**DOI:** 10.1186/1479-5868-7-83

**Published:** 2010-11-22

**Authors:** Weiwen Chai, Claudio R Nigg, Ian S Pagano, Robert W Motl, Caroline Horwath, Rod K Dishman

**Affiliations:** 1 Cancer Research Center of Hawaii, University of Hawaii, (1236 Lauhala Street), Honolulu, (HI 96813), USA; 2 Department of Public Health Studies, University of Hawaii, (1960 East West Road), Honolulu, (HI 96822), USA; 3 Department of Kinesiology and Community Health, University of Illinois - Champaign Urbana, (906 S. Goodwin Ave), Champaign, (IL 61820), USA; 4 Department of Human Nutrition, University of Otago, (33 Union Street), Dunedin, New Zealand; 5 Department of Kinesiology, University of Georgia, (330 River Road), Athens, (GA 30602), USA

## Abstract

**Introduction:**

High intake of fruit and vegetables and being physically active are associated with reduced risk of chronic diseases. In the current study, we examined the associations of physical activity, fruit and vegetable consumption, and TV/video watching (indicator for physical inactivity) with perceived quality of life (QOL) in a sample of free living adults.

**Methods:**

A cohort (N = 139) from a random, multi-ethnic sample of 700 adults living in Hawaii was evaluated at 3-month intervals for the first year and 6-month intervals for the second year. QOL was assessed from self-reports of mental or physical health at the end of the study.

**Results:**

Overall, the cohort participants appeared to maintain relatively constant levels of physical activity, fruit and vegetable intake, and TV/video watching. Physical activity was positively related to mental health (p-values < 0.05), but not physical health, at all time points regardless of participants' fruit and vegetable consumption and hours of TV/video watching. Neither mental nor physical health was associated with fruit and vegetable intake or TV/video watching.

**Conclusion:**

Our study supports that physical activity is positively associated with mental health. Fruit and vegetable consumption and TV/video watching may be too specific to represent an individual's overall nutritional status and physical inactivity, respectively.

## Introduction

High intake of fruit and vegetables and being physically active are associated with reduced risk of chronic diseases such as heart disease, diabetes, and cancers [1-5]. Despite interventions to increase physical activity in the general population, only 30% of US adults aged 18 years or older are sufficiently active during their leisure time according to recent surveys [6]. National campaigns such as 5-A-Day have increased awareness of the health benefits associated with fruit and vegetables; however, the increase in consumption of these foods has been relatively modest compared to the decrease in fat intake [7].

Quality of life (QOL), a conceptualization reflecting an individual's physical and mental well-being, has emerged as an important consideration in disease treatment and prevention. Research on QOL and physical activity has predominantly focused on elderly populations or populations with chronic diseases such as cardiovascular diseases, arthritis, pulmonary diseases, and cancer [1]. Although evidence consistently suggests a positive association between physical activity and QOL in these populations [8,9], the relation may not be reproducible in younger, disease-free individuals. In comparison to the physical activity domain, fewer studies have assessed the impact of fruit and vegetable intake on QOL and most of them were also conducted in diseased populations [10-14].

Physical inactivity has drawn far less research attention than physical activity; nevertheless, its adverse health effects may be as important as the beneficial effects of physical activity. It was reported that TV watching, a commonly used indicator of inactivity was associated with obesity [15,16]. Understanding behavioral patterns involving nutrition, physical activity, and inactivity and how they influence QOL is essential to public health as positive outcomes would provide the general public with a motivation to adopt healthy lifestyles, thereby reducing risk and incidence of chronic diseases. In the current study, we examined the associations of fruit and vegetables consumption, physical activity, and inactivity with QOL in a sample of free living adults in Hawaii.

## Methods

### Participants and procedures

This longitudinal, cohort study used a random sample of 700 adults (18 years or older) from Hawaii. A sub-sample of 139 (20%) participants who completed QOL survey at the end of the study was used for analysis (QOL cohort).

The detailed procedure was described previously [17]. In brief, the questionnaire was programmed into a computer assisted telephone interview system and participants were recruited using random digit dialing procedures. A qualified individual whose birthday was closest to the date of the phone call was asked to participate. A total of 700 adults were recruited and informed consent was obtained from the participants. The University of Hawaii Institutional Review Board approved all study procedures. At baseline (T-1), 3-month intervals (T-2, T-3, T-4, and T-5) for the first year and 6-month intervals (T -6 and T-7) for the second year, assessments (30-minute interviews) regarding participants' physical activities and nutritional behaviors were performed. A survey of QOL was sent out at the end of the study (T-7); 139 (20%) participants completed the survey.

### Measures of physical activity

Physical activity was assessed using the International Physical Activity Questionnaire (IPAQ) which records physical activity as hours and additional minutes of participation during the past 7 days in activities rated according to multiples of metabolic equivalents (METS). IPAQ assesses frequency and duration of walking (3.3 METS) and moderate (4.0 METS) and vigorous (8.0 METS) physical activity, appropriate for categorization of individuals as meeting public health guidelines for sufficient regular physical activity. The total weekly physical activity levels, expressed as MET_hr/wk, were calculated as the sum of walking and moderate and vigorous physical activity for the week. The IPAQ has acceptable measurement properties for monitoring population levels of physical activity among 18- to 65-year-old adults in diverse settings [18].

### Measures of physical inactivity

Physical inactivity/sedentary behavior was measured according to the amount of time (hours) each participant spent on watching TV/video on an average day as previously described [19]. Data suggest that TV/video watching far exceeds the time spent in any other leisure activity and represents the principal sedentary behavior in the United States [20].

### Measures of fruit and vegetable intake

The National Cancer Institute (NCI) Fruit and Vegetable screener was used in our study to assess the frequency and amount of consumption of 9 categories of fruits and vegetables (fruit, fruit juices, salad, beans, French fries, other potatoes, tomato sauce, vegetable soups, and other vegetables) over the previous month [21]. Computation of total daily servings of fruit and vegetables was described elsewhere [21]. This questionnaire provides estimated median daily servings of fruit and vegetables similar to those from 24-hour recalls [21].

### Measures of QOL

QOL was measured using a SF-12 Health Survey (SF-12). The SF-12 is a multipurpose short-form with 12 questions, all selected from the SF-36 Health Survey [22]. Scale sores were estimated for four health concepts (physical functioning, role physical, role emotional, and mental health) using two items each, whereas the remaining four (bodily pain, general health, vitality, and social functioning) were represented by a single item. All 12 items were used to calculate the physical (PCS) and mental (MCS) component summary scores by applying a scoring algorithm empirically based on the data of a US general population survey [22,23]. SF-12 was chosen in the current study since overall physical and mental health were the key outcomes of interest.

### Statistical analysis

Repeated-measures MIXED model was used to evaluate if physical activity, fruit and vegetable intake, or hours on TV/video watching were affected by time. Mean values at T-2, 3, 4, 5, 6 or 7 were compared to that at baseline (T-1).

Partial correlation was used to assess the associations of QOL outcomes (MCS or PCS) with physical activity, fruit and vegetable consumption, or TV/video watching. The basic model and the full model adjusted for ethnicity, gender, age (continuous), body mass index (BMI; continuous), and education (continuous) were tested. The results were similar for both models (data not shown). We also used General Linear Model (GLM) to examine the interactions between physical activity, fruit and vegetable intake, and TV/video watching in relation to MCS or PCS. T-test was used to assess the differences in the above three behavioral variables between the total random sample and QOL cohort at baseline.

In order to examine whether QOL outcomes were associated with certain behavioral patterns, we classified study participants into 9 profiles. The reason for this approach was to further explore the potential/suggestive associations which might not be detected by the above statistical analyses using continuous variables. The classification criteria were described as follow. A participant was classified as "average" if all his/her values (as means calculated from the 7 time points) for physical activity, fruit and vegetable intake, and TV/video watching were within 0.5 SD from the corresponding cohort means. The remaining categories (for "non-average" participants) were defined according to whether a participant's value for a certain behavioral variable was greater than 0.5 SD above or less than 0.5 SD below the cohort mean (e.g., high for physical activity, > cohort mean for physical activity + 0.5 SD; high for fruit and vegetable intake, > cohort mean for fruit and vegetable intake + 0.5 SD; low for TV/video watching, < cohort mean for TV/video watching - 0.5 SD). SAS software (SAS Institute, Cary, North Carolina) was used for all analyses. All tests were two sided, and *P *< 0.05 was considered statistically significant.

## Results

Table 1 shows baseline characteristics for the total random sample and QOL cohort. Although QOL cohort participants were more likely to be older, females, and Caucasians and less likely to be Pacific Islanders compared to the total random sample, there were no significant differences in baseline physical activity levels, fruit/vegetable consumption, and hours of TV/video watching between the two populations. It appeared that both populations had relatively high fruit and vegetable intakes at baseline (6.7 for QOL cohort and 7.4 for the total random sample).

**Table 1:** Selected baseline characteristics of the study participants

Characteristics	Total random sample (N = 700)	QOL cohort (N = 139)
Age (y), mean (SD)	47.0 (17.1)	55.3 (15.5)
BMI (kg/m^2^), mean (SD)	25.9 (5.6)	24.7 (4.7)
Education (y), mean (SD)	14.6 (2.8)	15.6 (2.9)
Median household income ($)	40-50,000	40-50,000
Gender, n (%)		
Male	256 (36.6)	36 (25.9)
Female	438 (62.6)	103 (74.1)
Ethnicity, n (%)		
Caucasian	261 (37.3)	59 (42.4)
Pacific islanders	155 (22.1)	20 (14.4)
Asian	218 (31.1)	43 (30.9)
Other	60 (8.6)	15 (10.8)
In excellent/very good/good/or fair health, n (%)	655 (95.0)	133 (95.7)
Physical activity (MET_hr/wk), mean (SD)	67.5 (66.2)	63.0 (59.2)
P = 0.46*		
Fruit and vegetable (servings/d), mean (SD)	7.4 (6.5)	6.7 (4.2)
P = 0.10*		
TV/video (hr/d), mean (SD)	2.5 (2.0)	2.3 (1.7)
P = 0.18*		

Note. QOL = quality of life; BMI = body mass index; MET = metabolic equivalent.

* T-test was performed to examine the differences between the total random sample and QOL cohort in weekly physical activity levels, daily fruit and vegetable consumption, and daily hours of TV/video watching at baseline.

Overall, physical activity levels, fruit and vegetable intake, and hours of TV/video watching remained relatively constant over the 2-year experimental period. However, physical activity levels were significantly lower at T-3 and T-6 than that at baseline. Participants also had significantly lower fruit and vegetable intakes at T-4 and T-6 compared to baseline (Table 2).

**Table 2:** Levels of physical activity, fruit and vegetable intake, and TV/video watching for participants of QOL cohort (N = 139) at the 7 time points

Time points	Physical activity	P*	Fruit and vegetable	P*	TV/video	P*
	(MET_hr/wk)		(serving/d)		(hr/d)	
Baseline (T-1)	63.0 ± 4.7		6.7 ± 0.4		2.3 ± 0.1	
3-month (T-2)	55.6 ± 4.7	0.11	7.0 ± 0.4	0.51	2.4 ± 0.1	0.44
6-month (T-3)	48.5 ± 4.8	0.001	6.7 ± 0.4	0.94	2.2 ± 0.1	0.46
9-month (T-4)	58.5 ± 4.7	0.35	5.8 ± 0.4	0.04	2.3 ± 0.1	0.97
12-month (T-5)	56.0 ± 4.8	0.13	6.0 ± 0.4	0.12	2.3 ± 0.1	0.94
18-month (T-6)	54.0 ± 4.8	0.048	5.5 ± 0.4	0.01	2.3 ± 0.1	0.95
24-month (T-7)	61.9 ± 4.8	0.87	6.6 ± 0.4	0.79	2.2 ± 0.1	0.50

Note: Values are presented as mean ± SE; QOL = quality of life. MET = metabolic equivalent

* P value for difference between T-1 (baseline) and T-2, T-3, T-4, T-5, T-6, or T-7 from repeated-measures MIXED model.

Increasing weekly physical activity levels was significantly associated with increasing MCS at all time points (T-1 to T-7; P-values < 0.05). There were no significant associations between MCS and daily fruit and vegetable consumption. No significant associations were also observed between MCS and daily hours of TV/video watching except for T-3 and T-7, where an inverse association was observed. PCS was not associated with physical activity, fruit and vegetable intake, or hours of TV/video watching (Table 3). There were no significant interactions between physical activity, fruit and vegetable consumption, and TV/video watching in relation to MCS or PCS (P-values > 0.05).

**Table 3:** Correlations of physical activity, fruit and vegetable intake, and TV/video watching with QOL outcomes at the 7 time points*

Time points		MCS		PCS	
		**r**	**P**	**r**	**P**

Baseline	Physical activity	0.21	0.02	0.01	0.96
(T-1)	Fruit and Vegetable	-0.02	0.81	-0.02	0.79
	TV/video	-0.00	0.99	0.02	0.82
					
3-month	Physical activity	0.21	0.02	0.06	0.50
(T-2)	Fruit and Vegetable	0.02	0.84	0.03	0.75
	TV/video	-0.14	0.14	0.04	0.66
					
6-month	Physical activity	0.21	0.02	0.08	0.37
(T-3)	Fruit and Vegetable	0.09	0.28	-0.07	0.42
	TV/video	-0.24	0.008	0.14	0.12
					
9-month	Physical activity	0.25	0.006	0.09	0.30
(T-4)	Fruit and Vegetable	-0.06	0.50	0.07	0.41
	TV/video	-0.01	0.96	0.07	0.45
					
12-month	Physical activity	0.21	0.02	0.04	0.70
(T-5)	Fruit and Vegetable	0.13	0.14	-0.06	0.51
	TV/video	-0.02	0.87	-0.07	0.45
					
18-month	Physical activity	0.24	0.009	0.12	0.21
(T-6)	Fruit and Vegetable	0.12	0.19	0.03	0.71
	TV/video	-0.18	0.05	-0.06	0.49
					
24-month	Physical activity	0.24	0.01	0.05	0.56
(T-7)	Fruit and Vegetable	0.09	0.35	-0.04	0.65
	TV/video	-0.21	0.02	-0.06	0.55

Note. QOL = quality of life; MCS = mental component summary for SF-12; PCS = physical component summary for SF-12; r = correlation coefficient

* Partial correlations were performed and results were adjusted for ethnicity, gender, age, body mass index, and education.

The QOL outcomes for the 9 behavioral profiles were listed in Table 4. We did not perform statistical analyses due to small sample size for certain behavioral profiles. Consistent with our overall findings, participants who had average or above average physical activity levels were also characterized by higher MCS scores regardless of their status of fruit and vegetable consumption and/or TV/video watching.

**Table 4:** QOL outcomes for behavioral profiles

Behavioral profiles*				Behavioral parameters mean (SD)			QOL measures mean (SD)	
**PA**	**FV**	**TV/video**	**N**	**PA** **(MET_hr/wk)**	**FA** **(serving/d)**	**TV/video** **(hr/d)**	**PCS**	**MCS**

low	low	low	11	23.6 (8.8)	3.4 (1.0)	1.7 (0.8)	49.9 (9.1)	44.1 (10.4)
low	low	high	13	15.6 (8.4)	2.7 (0.9)	3.7 (1.7)	48.4 (11.0)	48.7 (10.6)
low	high	low	16	21.3 (9.1)	7.8 (2.0)	1.6 (0.7)	44.5 (11.8)	49.0 (10.3)
low	high	high	14	23.3 (12.3)	7.1 (1.6)	3.7 (1.1)	48.7 (10.9)	45.6 (12.4)
high	high	high	10	134.8 (57.2)	7.8 (2.8)	3.6 (0.8)	47.0 (9.3)	54.5 (6.7)
high	high	low	32	92.6 (38.9)	8.6 (3.0)	1.3 (0.8)	48.3 (8.3)	53.7 (6.9)
high	low	high	6	80.1 (56.0)	4.4 (0.5)	4.1 (0.5)	51.0 (4.3)	54.2 (7.1)
high	low	low	3	44.9 (6.0)	3.0 (0.3)	0.6 (0.5)	46.9 (8.8)	54.6 (3.6)
Average^†^			34	54.6 (14.9)	5.8 (1.6)	2.0 (1.8)	50.4 (8.6)	51.9 (8.3)

Note. QOL = quality of life; PA = physical activity; FV = fruit and vegetable intake; PCS = physical component summary for SF-12; MCS = mental component summary for SF-12; MET = metabolic equivalent.

* 'high' refers to the participant's value for PA, FV or TV/video was greater than 0.5 SD above the cohort mean for the respective variables; 'low' refers to the participant's value for PA, FV, or TV/video was less than 0.5 SD below the cohort mean for the respective variables.

^† ^Participants were classified as 'average' if their values for PA, FV, and TV/video were all within 0.5 SD from the corresponding cohort means.

## Discussion

Our results indicated that physical activity was predictive of positive mental health irrespective of participants' other behaviors such as fruit and vegetable intake and TV/video watching. This observation is in agreement with other prospective studies that showed physically active adults had lower risk of mental distress than inactive adults when measures like the SF-12, including its parent measure, the SF-36, are used [1,24]. However, those studies did not uniformly adjust for diet or sedentary behaviors that might confound the association between perceived distress and physical activity [1,24]. The results are plausible given the large literature from prospective cohort studies and randomized controlled trials that regular physical activity is associated with reduced symptoms of depression and anxiety, which represent the main content areas of the mental health scale of the SF-12. Although limited, there is emerging evidence to support neurobiological mechanisms whereby physical activity can reduce feelings of depression or anxiety by positive influences on the central nervous system [25,26] Alternatively, physical activity may also influence mental health through social interactions due to the mutual support and social relationships that are provided when engaging in physical activity with others.

No associations were found between participants' physical health and levels of physical activity. Previous research conducted in elderly or chronically diseased populations demonstrated strong evidence that exercise improved individuals' physical conditions [1]. It is suggested that older adults and those with chronic diseases tend to have poorer physical health which creates challenges and requires specific needs concerning physical activity [1]. Consequently, results obtained from these populations may not be generalized to populations with different age and physical conditions. The mean participant age in our study was 55.3 ± 15.5 years. Approximately 96% reported that their health was excellent, very good, good, or fair. Thus, the lack of association observed in the current study could be partially attributed to our younger and healthier study population (essentially, a ceiling effect).

In our study, neither physical nor mental health was associated with fruit and vegetable consumption. Similar to physical activity domain, previous studies demonstrating positive associations between nutrition and QOL were also predominately conducted in diseased populations. In our study, the average intakes of fruit and vegetables at each time point were high, all exceeding the currently recommended 5 servings/day. As a consequence, the ability to detect the potential associations may be limited due to the high intake (another ceiling effect). Furthermore, food and nutrition can affect people's lives through many ways. Instead of focusing on individual dietary component, we need to consider the roles of other factors such as meal preparation, additional food groups, dinning environment, social interaction, and cultural aspects of food and diet.

Overall, TV/video watching was not associated with mental or physical heath in the current study. The inverse association with MCS observed at T-3 and T-7 is intriguing and requires further investigation. It was suggested that depression and sedentary lifestyle have bidirectional relationships [27]. Another study by Patten et al. [28] showed that major depressive episodes were associated with an increased risk of transition from an active to an inactive pattern of activity. However, physical inactivity in the above studies was measured according to participant's physical activity levels or daily energy expenditure instead of quantifying the time spent in sedentary behaviors such as TV/video watching.

There were several limitations to the current study. First, although we used validated self-reported measures, objective indicators may provide more accurate evaluations of behaviors. Second, QOL was only assessed at the end of the study (T-7), resulting in a low response rate for the survey (20%). However, in light of the evidence that participants maintained relatively constant levels of the behavioral parameters and the key variables from QOL cohort were not considerably different from the entire sample, we presume that our findings would persist over the 2-year study period. Finally, we did not measure other sedentary behaviors such as work-related physical inactivity which could be relevant to QOL outcomes.

In conclusion, the results from this longitudinal study indicated physical activity, superior to fruit and vegetable consumption and physical inactivity, is a main player influencing individual's mental health. Our findings are novel and support future investigations in a larger study involving repeated measurements of QOL and multi-behavioral frame work.

## Abbreviations

QOL: Quality of life; SF-12: Short Form Health Survey; MCS: mental component summary for SF-12; PCS: physical component summary for SF-12; MET: metabolic equivalent; SD: standard deviation; SE: standard error.

## Competing interests

The authors declare that they have no competing interests.

## Authors' contributions

All of the authors made substantial contributions to the study concept and design or analysis and interpretation of the data and have reviewed and approved the final manuscript for submission. Specifically, WC designed the analysis, analyzed the results, and was the primary author of every section of the text. ISP aided in the design of the analysis and performed much of the initial statistical analyses. CRN, RWM, CH, and RKD were instrumental in the design of the study, and commented on and approved the manuscript.
